# Association study of single nucleotide polymorphism rs165599 of *COMT *gene, with schizophrenia and bipolar mood disorder in the south-west of Iran

**Published:** 2015-06

**Authors:** Parisima Behbahani, Seyed Reza Kazemi-Nezhad, Ali Mohammad Foroughmand, Leila Ahmadi

**Affiliations:** Department of Genetics, Faculty of Science, Shahid Chamran University, Ahvaz, Iran

**Keywords:** Bipolar mood disorder, *COMT *gene, rs165599, Schizophrenia

## Abstract

Linkage studies and epidemiological findings indicate that some possible genes in schizophrenia (SCZ) and bipolar mood disorder (BPD) are common. Numerous evidences for linkage of two diseases on chromosome 22 have been found. These findings suggest that one or more genes in the 22q11.21 region may be involved in the development of both disorders. In the present case-control study, association between the mentioned disorders and a genetic polymorphism (rs165599) of catechol O- methyltransferase (*COMT*, OMIM: 116790) was studied. Here 100 BPD patients, 100SCZ patients, and 100 healthy controls were included in the study. The samples were matched in terms of gender and ethnicity. Statistical analysis showed that there was a significant association this polymorphism and risk of SCZ. The AG (OR=7.41, 95% CI: 3.21-17.1, P<0.001) and GG genotypes (OR=13.9, 95% CI: 5.61-34.4, P<0.001) increased the risk of SCZ compared with the GG genotypes. The AG (OR=14.3, 95% CI: 4.16-49.4, P<0.001) and AA genotypes (OR=54.2, 95% CI: 15.3-191, P<0.001) significantly associated with the risk of BPD. The risk of SCZ (*x*^2^=37.4, P<0.001) and BPD (*x*^2^=66.2, P<0.001) significantly increased as a function of numbers of the A allele. The present study revealed that this polymorphism associated with risks of SCZ, and BPD.

## INTRODUCTION

Psychiatric disorders are the third largest health problem in developed countries. Schizophrenia (SCZ) and bipolar mood disorders (BPD) are samples of chronic disorders that are costly and heavy burdens for society and family [1]. Both SCZ and BPD are important psychiatric disorders. The genetic and environmental causes and factors are involved in SCZ incidence. The symptoms of SCZ include delusions, hallucinations, disorganized speech and behavior or catatonic [2]. BPD is a chronic condition that can lead to severe consequences in family, community and professional work [3]. BPD, which is also called manic-depression disorder, is characterized by alternating episodes of mania or hypomania and depression. The frequency of affected people is about 1-2% of the world population [4-6].

Linkage studies and epidemiological findings led to the suggestion that some genes associated with BPD and SCZ are common [7]. Evidences for linkage to chromosome 22 have been found in both diseases. Based on these findings, one or more genes in 22q11.21 region may be involved in the occurrence of these disorders [8].

Genetic studies have revealed that first- or second-degree relatives of individuals with SCZ or BPD are at high risk for these two disorders. Recent studies emphasize that increased risk of mental illness in relatives of probands with SCZ, is not limited to SCZ [6]. In the other word, in the relatives of SCZ probands there is an increased risk of BPD [9], justifying the assumption that the same genes may cause both diseases. In fact, several candidate genes for SCZ, including; *COMT*, *G72 *and *DISC1*, may also be associated with BPD [10, 11]. Catechol O-methyltransferase (*COMT*, OMIM: 116790) gene (located on human chromosome 22q11) has been studied in several mental disorders associated with SCZ [12]. In addition, many studies have shown that individuals with Velocardiofacial syndrome (VCFS) due to 22q11 deletion may show some types of mental disorders [13]. Accordingly, one or several removed genes in the regions of *COMT *may be involved in their mental disorders. The COMT protein affects the dopamine destruction, which has been proved as a potent neurotransmitter in many studies [14]. The rs165599 polymorphism is in the 3'UTR, reduces the mRNA expression [8, 15]. COMT is one of the enzymes that break down catecholamines such as dopamine. The human chromosome 22q11, is a candidate region for SCZ and BPD [16-18]. The main aim of this study is investigating the association of polymorphism (rs165599) of *COMT *with risk of SCZ and BPD.

## MATERIALS AND METHODS

**Clinical samples collection**: Based on DSM-IV-TR standard, 100 blood samples of patients with SCZ (67 men and 33 women) and 100 patients having BPD (47 men and 53 women) on their admissions in Golestan hospital (Ahvaz, Iran) were collected. The patients were evaluated by at least two psychiatrists during their admission. All the patients were treated with anti-psychotic and/or mood stabilizers drug during evaluation. The control group consisted of 127 non-relative (70 males and 57 females) individuals whom were screened in two steps. Firstly, they were interviewed using a questionnaire which showed that the individual or his/her first and second degree relatives are not at least one of the following: history of referral to a psychiatrist or psychologist, history of consuming psychiatric drugs, psychiatric hospitalization history, history of suicide attempts, and history of substance abuse or dependency.

Secondly, the screening was completed by GHQ (General Health Questionnaire). These criteria resulting in only the healthy subjects were included in the study. Healthy control, SCZ patients and BPD patients subsequently had mean±SD age 37.6±9.6, 36.9±10.2 and 34.4±11.2.

**DNA extraction and genotyping analysis: **Genomic DNA was prepared from blood samples through use of the Diatom DNA extraction kit (IsoGene, Moscow, Russia). The concentration of the genomic DNA was determined in an Uvisco UV/V-1200 Symantec (Switzerland) spectrophotometer. For the amplification of the DNA following primers were used: SNP rs165599-F: 5'-CAC AGT GGT GCA GAG GTC AG-3' and R:5'-TCA TCA CCA TCG AGA TCA ACC-3'. 10 µl of PCR product was digested with 3 µl *Msp*I (*Hpa*II, from Fermentas, Germany), in a total reaction volume of 15 ml containing 2 µl of commercial buffer and were subjected to the following cycling protocol: 95°C for 10 min; then 94°C for 30 sec, 60°C for 30 sec, and 72°C for 30 sec, for 35 cycles; followed by 72°C for 10 min. Fragments were separated on 3%agarose gel stained with safe stain (Bio-Rad, Germany).

**Statistical analysis: **The results were analyzed using the statistical software SPSS16, logistic regression. The results evaluated at significance level P<0.05.

## RESULTS AND DISCUSSION

Table 1 shows the genotypic frequencies of the study polymorphism of *COMT *in the BPD, SZC and control groups. Control subjects were at Hardy-Weinberg equilibrium (*x*^2^=3.28, df=1, P=0.069). Statistical analysis showed that there was a significant association this polymorphism and risk of SCZ. The AG (OR=7.41, 95% CI: 3.21-17.1, P<0.001) and GG genotypes (OR=13.9, 95% CI: 5.61-34.4, P<0.001) increased the risk of SCZ compared with the GG genotypes. Comparison between BPD and control subjects revealed same associations. It means that the AG (OR=14.3, 95% CI: 4.16-49.4, P<0.001) and AA genotypes (OR=54.2, 95% CI:15.3-191, P<0.001) significantly associated with the risk of BPD. The risk of SCZ (*x*^2^=37.4, P<0.001) and BPD (*x*^2^=66.2, P<0.001) significantly increased as a function of numbers of the A allele.

**Table 1:** Genotypic distribution of the rs165599 polymorphism in cases and control groups

**Genotypes**	**Controls**	**SCZ**	**OR**	**95% CI**	**P**	**BPD**	**OR**	**95% CI**	**P**
GG	57	8	1.0	-	-	3	1.0	-	-
AG	49	51	7.41	3.21-17.1	<0.001	37	14.3	4.16-49.4	<0.001
AA	21	41	13.9	5.61-34.4	<0.001	60	54.2	15.3-191	<0.001

It is known that common functional polymorphism Val/Met *COMT *gene, affects the enzyme activity and physiology of human cortex. High activity allele, Val, increases SCZ risk through effects on early information processing by dopamine. The polymorphism of Val/Met has been studied by many researchers [19, 20]. Shifman et al. [8] observed the association between SCZ and rs4680. But Lajin and colleagues [21], showed no association between SCZ and rs4680. Shifman et al. at 2004 showed lack of association of rs4680 with BPD while results of Mynett-Johnson [22] and colleagues in1998 demonstrated an association between rs4680 and BPD. Studies showed differences in the results and raises the possibility of other polymorphisms affection on the enzyme activity.

As a result other SNPs in the *COMT *gene, especially one SNP that is located in the 3' untranslated region close to exon 1 of MB-*COMT *isoforms; rs165599 have reported that expression of *COMT *is associated with risk of SCZ [23, 24]. Shifman also showed the association between rs165599 and the SCZ disorder [8]. The association study of Shifman and his team show a positive correlation between rs165599 and BPD [8]. However, polymorphism rs165599 in the 3'UTR by affecting transcription, mRNA stability, and translation efficiency, reduces mRNA expression [9, 15].

Despite many reported association between *COMT *gene, SCZ and BPD, there are some study with different results. These conflicting results may be interpreted using following reasons:

- One of the most important causes is population differences.

- Another possible cause is related to how subjects are selected. Psychiatric interview or various test are methods of detection of mental disorder.

In the present study the GHQ test has been applied to diagnose healthy individuals, provided that they own no first or second degree relatives who have a history of mental illness no drug addiction, and no inpatient psychiatric drugs, then association studies on polymorphism of *COMT *gene with SCZ and BPD have been done. The rs165599 polymorphism of *COMT *has not yet been widely studied. The present study revealed that this polymorphism associated with risks of SCZ, and BPD.

## Notes

**Acknowledgements: **This work is supported by the Research and technology deputy of Shahid Chamran University of Ahvaz, Iran. Thanks to the sincere cooperation of the staff of the psychiatric ward in Golestan hospital and the Blood Transfusion Organization of Khuzestan province.
